# A risk prediction tool for colorectal cancer screening: a qualitative study of patient and provider facilitators and barriers

**DOI:** 10.1186/s12875-020-01113-0

**Published:** 2020-02-26

**Authors:** Marianne S. Matthias, Thomas F. Imperiale

**Affiliations:** 1 Center for Health Information and Communication, Roudebush Veterans Affairs Medical Center, 1481 W. 10th Street 11H, Indianapolis, IN 46202 USA; 2 Department of Medicine, Indiana University School of Medicine, Indianapolis, IN USA; 3 Regenstrief Institute, Inc, Indianapolis, IN USA; 4 Department of Communication Studies, Indiana University-Purdue University Indianapolis, Indianapolis, IN USA; 5 Richard M Fairbanks School of Public Health, Indiana University-Purdue University of Indianapolis, Indianapolis, USA

**Keywords:** CRC screening, Risk prediction tools, Implementation, Qualitative research, Cancer prevention

## Abstract

**Background:**

Despite proven effectiveness of colorectal cancer (CRC) screening, at least 35% of screen-eligible adults are not current with screening. Decision aids and risk prediction tools may help increase uptake, adherence, and efficiency of CRC screening by presenting lower-risk patients with options less invasive than colonoscopy. The purpose of this qualitative study was to determine patient and provider perceptions of facilitators and barriers to use of a risk prediction tool for advanced colorectal neoplasia (CRC and advanced, precancerous polyps), to maximize its chances of successful clinical implementation.

**Methods:**

We conducted qualitative, semi-structured interviews with patients aged 50–75 years who were not current with CRC screening, and primary care providers (PCPs) at an academic and a U.S. Department of Veterans Affairs Medical Center in the Midwest from October 2016 through March 2017. Participants were asked about their current experiences discussing CRC screening, then were shown the risk tool and asked about its acceptability, barriers, facilitators, and whether they would use it to guide their choice of a screening test. The constant comparative method guided analysis.

**Results:**

Thirty patients and PCPs participated. Among facilitators were the tool’s potential to increase screening uptake, reduce patient risk, improve resource allocation, and facilitate discussion about CRC screening. PCP-identified barriers included concerns about the tool’s accuracy, consistency with guidelines, and time constraints.

**Conclusions:**

Patients and PCPs found the risk prediction tool useful, with potential to increase uptake, safety, and efficiency of CRC screening, indicating potential acceptability and feasibility of implementation into clinical practice.

## Background

Colorectal cancer (CRC) is the third most common cancer in the U.S. and the second most common cause of cancer mortality [1]. While screening for CRC is effective and cost-effective, 30–35% of the screen-eligible U.S. population is not current with screening [2]. Colonoscopy is the most widely known and frequently used test; however, several alternative, less invasive tests are available. Providing patients with a choice of screening test increases screening uptake [3]. Given the absence of evidence for superiority of any single screening strategy, the U.S. Preventive Services Task Force recommends that providers and patients engage in shared decision-making when choosing a screening test [4]. Components of a shared decision-making exchange should include the tradeoffs among screening tests, patient preferences, and patient risk for CRC or advanced neoplasia (i.e., the combination of CRC and advanced, precancerous polyps).

Understanding risk for CRC, advanced neoplasia, or both may help patients and providers choose an appropriate screening test. While several decision aids and risk prediction models are available [5–14], they are not used in clinical practice for several reasons, including lack of independent validation, limited discrimination, uncertain generalizability, and lack of clinical integration. In prior work, we derived and validated a simple five-variable risk prediction tool for advanced colorectal neoplasia (the combination of CRC and advanced, precancerous polyps) that overcomes many limitations of other scoring systems in that it uses easily- and reliably-measured variables (age, sex, CRC in a first-degree relative, cigarette smoking, and waist circumference) to estimate the risk of advanced neoplasia. The tool generates a score and has four risk strata ranging from very low to high risk [5]. The risk strata may be linked to specific screening tests, but this is not an intrinsic part of the tool.

A key element to the tool’s ability to increase screening uptake is patient and provider perceptions of facilitators and barriers to its use. Although the tool has not yet been formally tested to demonstrate higher screening uptake, there is increasing recognition that, to address the substantial lag between demonstration of effectiveness and implementation into clinical practice, attention to implementation issues, such as facilitators and barriers, must occur early in the research process [15]. This strategy for exploring such personalized cancer screening was recommended at a recent National Cancer Institute-sponsored symposium [16].

The purpose of the current study was to identify patients’ and primary care providers’ (PCPs) perceptions of facilitators and barriers to use of the tool in clinical practice by conducting qualitative interviews with patients eligible for CRC screening and with PCPs.

## Methods

This study was approved by the Indiana University Institutional Review Board and by the Research and Development Committee of the Richard L. Roudebush VA Medical Center, Indianapolis, IN. All study participants provided written informed consent to participate in the research and allow their de-identified data to be used.

### Study design

We used a qualitative approach for this study because such an approach is useful for newer areas of inquiry, such as understanding a new tool, and for eliciting rich, detailed experiences of participants [17, 18]. Individual interviews were conducted in an effort to obtain detailed, honest answers that are not influenced by the opinions of others or social desirability. Qualitative interviews were conducted with patients and PCPs from an academic and a U.S. Department of Veterans Affairs (VA) medical center in Indianapolis, IN, from October 2016 to March 2017.

### Participants

We used criterion sampling [19] to select patients within two hospital systems. Patients were eligible if they were aged 50–75 years and not current with CRC screening, meaning no colonoscopy within the previous 10 years or no fecal immunochemical test (FIT) within the previous year. All PCPs were eligible and were sampled using convenience sampling. All patient and provider participants received a gift card.

### Recruitment

Potentially eligible patients were identified through both hospitals’ electronic medical records. With permission from their PCPs, patients were mailed letters explaining the study, followed by a phone call. Eligible, interested patients were scheduled for an interview. Eligible PCPs were contacted by e-mail; those interested in participating were scheduled for an interview.

### Interviews

Personnel not involved in tool development conducted face-to-face interviews, after participants signed an informed consent and had the opportunity to ask questions. Interviews lasted an average of 30 min (range: 15–40 min) and were audio-recorded, transcribed, checked for accuracy, and de-identified. In the interview, participants were asked about experiences discussing CRC screening, using questions such as “Which tool(s) do you recommend to patients who need screening?” (PCPs) or, “Which test(s) did your primary care provider recommend to you for CRC screening?” (patients). Next, the interviewer showed and explained the risk prediction tool. Interviewers asked PCPs about their impression of the tool, including the likelihood of using it and potential barriers and facilitators to its use; patients were asked about their impressions, including whether they would use it to guide their choice of a screening test. Interviews continued until saturation was reached (i.e., additional data no longer evoked new theoretical insights or illuminated new properties in the codes) [18]. The interview guide is included in the Additional file 1.

### Data analysis

The analytic team comprised a communication scientist and a gastroenterologist. The constant comparison method, which is rooted in grounded theory, guided analysis [18, 20]. This method consisted of open and focused coding. During open coding, analysts read all transcripts to gain an understanding of the data and variation across participants. Emergent themes (i.e., codes) were identified and iteratively refined through multiple readings and discussions. Once codes were stable and consistent, focused coding began; authors applied codes derived in phase 1 to all transcripts, meeting regularly to compare coding and ensure consistency. During analysis, authors practiced reflexivity (i.e., reflecting on one’s knowledge and background and how this may influence interpretation of data), and sought negative cases that might lead to alternative interpretations of the data [18, 21, 22]. Discrepancies were resolved by consensus.

## Results

Thirty participants (15 patients, 15 PCPs) participated. Seven patients and eight PCPs (6 general internists, 2 nurse practitioners) were from the VA medical center. The remaining patients (*n* = 8) and PCPs (*n* = 7; all general internists) were from the academic medical center.

### Demographics

Mean (SD) patient age was 59.8 (7.4) years; 47% were women. Forty percent were non-Hispanic White; 60% were non-Hispanic Black. PCPs’ mean (SD) age was 46.5 (9.3) years; 47% were women; 80% were White.

### Facilitators

Participants identified 4 facilitators for using the risk prediction tool in practice: 1) use of risk tools is consistent with current practice; 2) the tool has potential to increase screening rates; 3) the tool could lead to improved patient safety and resource allocation, and; 4) the tool could facilitate discussion about CRC screening.

#### Consistent with current practice

PCPs believed using a risk prediction tool in CRC screening was aligned with current screening practices in other areas, including cholesterol management and breast cancer screening:

“It would follow along with risk and recommendations for cholesterol medication” (PCP203).

The tool “is similar to using the Gail model when screening for breast cancer” (PCP407).

#### Potential to increase CRC screening rates

Because the tool does not yield a colonoscopy recommendation for all patients, PCPs noted the ability to emphasize screening options—potentially leading to greater uptake of testing, particularly when patients are reluctant to undergo colonoscopy.

“Everybody’s looking for the out…not having to go through the colonoscopy. They’re going to go to the point of least resistance…well, that’s a lot easier than drinking that gallon of junk” (PCP202).

“I think somebody who’s adamantly opposed to a colonoscopy but willing to do a stool test, it would help…it certainly could increase how many people do stool tests” (PCP403).

The tool “might get more people in the door and agreeable to screening” (PCP401).

One PCP noted increased uptake would likely translate to catching more cancers:

“Stool testing is great for catching colon cancer. At this point I just want to screen more people.” (PCP402).

Patients corroborated this idea of increased screening uptake. One patient shared that if given the choice, he would opt for FIT over colonoscopy, saying, “I’ve heard colonoscopy was kind of painful, or could be…I’ve heard…some scary stories.” He further stated that if his risk category were appropriate for FIT, “that makes me a little more eager to get it scheduled, because I don’t have to dread the pain. As much as I wanted to have the relief of knowing, I really didn’t want to go through the pain” (PT302).

One patient cited time savings of FIT over colonoscopy: “Five minutes [for FIT] compared to hours. I most definitely would do it” (PT103). Another patient said simply of FIT, “It beats the other option” (PT305).

Patients pointed out a barrier to testing that PCPs did not mention: patient cost. Patients said they might be more likely to get tested if offered FIT because it is more affordable. One patient said that FIT “sounds like it’s more in the budget” (PT305).

Even providers with less confidence in FIT noted that some test is better than no test: “Even if FIT isn’t the best test, at least I get some result. [With] colonoscopy, I’m not sure I get the same compliance. So you’re sort of weighing compliance versus sensitivity” (PCP205).

#### Safety and resource allocation

PCPs said that encouraging patients toward FIT, when appropriate, had advantages, including safety:

“We would target the test according to the risk, so that seems more…appropriate...more rational, and maybe even a little bit safer, if we could avoid doing colonoscopies on patients with very low risk. It seems like a better balance of risk and benefit” (PCP201).

One PCP saw cost savings as an advantage: “What appeals to me is that there is additional data that would indicate that maybe we could use less risky, cheaper modalities to have similar efficacy in screening” (PCP202).

Improved resource allocation was also cited as a major potential advantage:

“It might help get people who are low and very low risk, to put them into the FIT test, and that will ease some of the workload for colonoscopies…And probably most people would prefer that anyway, so it’s easier to get it done, and we probably would have better resource utilization” (PCP206).

Pragmatically, several PCPs noted that in terms of quality measures, PCPs receive the “same points” for FIT or colonoscopy screening.

#### Generating discussion about CRC screening

PCPs indicated that the tool could help to facilitate conversation about CRC screening.

“It cannot just help the patients, but help the providers with the conversation about cancer screening” (PCP403).

Another PCP noted that the tool would be “a jump off point…You always want to have as many tools in your tool box as possible, so I would use it…to sway the discussion if needed. But also just show them the data so they know the easiest and best thing for them” (PCP202).

One provider noted the tool would save time during clinic visits: “It would be wonderful because…you wouldn’t have to talk about colonoscopy at all [with low-risk patients]. You could concentrate on people that really need colonoscopies” (PCP203).

Another provider indicated that the tool could serve as a reminder to recommend the most appropriate test:

“I know high-risk patients need colonoscopy, but…maybe it would help me not push my very low risk and low risk patients to get a colonoscopy, to tell them that the stool test is fine” (PCP402).

Some PCPs indicated that the tool could serve as a means to persuade patients to get screened—particularly because it generates an individualized risk score.

“I have found that true in the other [tools] that I use when I can show them their cholesterol and things like that. When I can show them that number: You’re at 10% risk in the next 10 years of having a cardiovascular event, and you should be on atorvastatin. [And they say] ‘Oh, ok’. I think it helps to visualize those things for patients” (PCP404).

The notion of visualization and having a “hard number” to show patients their risk was echoed by a number of PCPs:

“If you can show patients a risk score, it can help to persuade them” (PCP404).

“Sometimes visuals are more powerful than words” (PCP208).

“You could concentrate on people that really need colonoscopies and tell them the statistics, and have some hard data to kind of lead them [to colonoscopy]” (PCP203).

In a general reaction to the tool, one PCP told us, “I don’t see a lot of down sides to something this simple” (PCP205).

### Barriers

Patients generally did not identify barriers to the tool’s use. However, some PCPs expressed reservations about use in clinical practice. Concerns were related to 1) skepticism about the tool’s accuracy, 2) consistency with guidelines, 3) not having time to use the tool.

#### Skepticism about the tool’s accuracy

Some providers questioned the data used to develop the tool. Some said they would like to “see the references” (PCP205) and “know where the numbers came from” (PCP407). One PCP said that it would be helpful to know more about how the tool was developed and why the risk factors were chosen: “I think it would give [me] a little more faith in the scoring system” (PCP403).

It appeared difficult for some providers to move past the belief that colonoscopy should always be the first screening choice. One provider asked of the tool, “What’s that based on since colonoscopy is the gold standard?” (PCP204) Another PCP questioned whether “it works as well as a colonoscopy” (PCP207).

One patient echoed these sentiments: “Nothing is more definite than a colonoscopy. I mean going up in there and just being able to see is probably 100%.” (PT301) Most patients, however, said that they would be comfortable if the risk tool indicated a stool test instead of a colonoscopy.

#### Consistency with guidelines

Some PCPs pointed out that the risk prediction tool is not written into current guidelines, which made them hesitant to use it. One PCP stated that the tool could be useful “only if it falls in line with current accepted guidelines” (PCP204). Another provider noted, “It’s not incorporated into any of the guidelines. So even though it makes clinical sense, I would probably like to see…guidance from national organizations” (PCP401).

Perceived inconsistency with guidelines seemed related, in some cases, to legal concerns about missing a cancer. One PCP said, “As a provider, I wouldn’t want to put myself in some legal limbo…without knowing this is a recommended thing by accepted agencies” (PCP204). However, other providers dismissed this as a risk: “Yeah, we miss a cancer diagnosis, but it’s not because of the tool. It’s because of time or not doing [a test]” (PCP208). Another provider pointed out, “I always have my own judgment to override the tool” (PCP207).

#### Not having time to use the tool

Some PCPs were concerned about having time to discuss the tool. One provider thought that time was especially likely to be an issue if a patient had “more acute” issues (PCP203). Another described time as the “main barrier” to using the tool (PCP208). One PCP lamented that CRC screening is essentially competing with screening for various other health conditions: “It’s so tough, because there are also the same kinds of risks for, like, breast cancer…I’ve got 15 minutes, and I don’t always have time to figure out how much risk is in each [disease]…This is additional time and effort” (PCP406).

Another time-related concern had to do with gathering necessary information for the tool. Some providers were concerned about obtaining information such as waist circumference. One PCP said, “If I’m going to search through the medical record to get all these five risk factors—two are easy; three are going to take some work. So if that’s going to take me ten minutes and my patient is only seeing me for 15 minutes anyway. You know, I might not do it.” (PCP201).

Finally, some PCPs were concerned that it would take too much time to explain the tool—that patients would have difficulty understanding how the tool works, particularly concepts such as cigarette pack-years. One provider expressed skepticism as follows:

“It’s a little hard to say if they would understand…without explanation... I don’t have a sense of the extent to which patients could do that” (PCP201).

In contrast, the following PCP thought that even patients with low health literacy could understand it:

“A lot of my patients have low health literacy…I think that this [tool] is pretty straightforward, especially if I had a piece of paper or something on the computer to show them their risk. I think they would understand that” (PCP402).

Patients overwhelmingly indicated that they understood the tool, describing it as “very easy” (PT101) and “self-explanatory” (103). One patient (PT301) indicated the concept of pack-years was difficult to understand.

PCPs expressed a preference to see the tool in practice in either of the following formats: 1) built into the EMR with pre-populated variables (age, sex), completing variables absent from the EMR (waist circumference), followed by seeing the patient’s risk estimate for advanced neoplasia, or; 2) a hard copy “cover” sheet with patient-specific variables along with computed risk for advanced neoplasia.

## Discussion

A third of patients eligible for CRC screening are not current with screening [2]; consequently, methods are needed to help increase uptake, with the ultimate goal of catching and preventing CRC cancer. Further, as we are in the era of precision medicine, some have advocated for tailoring CRC screening based on risk. While precision cancer screening is believed to hold promise for increasing uptake of cancer screening in general, little information is available on optimizing its implementation into clinical practice, including patient and provider attitudes towards risk prediction tools [16]. The purpose of this study was to understand patients’ and PCPs’ perceptions of facilitators and barriers to use of a new risk prediction tool designed to help providers guide patients toward the most appropriate screening method based on their individual risk for advanced colorectal neoplasia. Addressing barriers and supporting facilitators of the tool is expected to increase the likelihood of successful clinical implementation [15].

PCPs and patients due for CRC screening were presented with a risk prediction tool and asked about their willingness to consider using the tool in clinical practice and to direct their screening, respectively. Through these interviews, we identified 4 facilitators and 3 barriers to its use. The four facilitators were: 1) consistency with current practice in other areas; 2) potential to increase uptake of screening; 3) a better balance of benefit, risk, and resource allocation, and; 4) facilitating the discussion about CRC screening between patients and providers. The three barriers were: 1) concern about the tool’s accuracy; 2) no endorsement of the tool as of yet from guidelines organization, and; 3) lack of time to use the tool in real-time clinical practice.

The devised risk prediction tool provides points for age, gender, family history of CRC, cigarette smoking, and waist circumference, resulting in risk categories of very low-, low-, intermediate-, and high-risk for advanced neoplasia, with respective risk ranges of 1.65–1.92%, 3.31–4.88%, 8.26–9.93%, and 22.3–24.9%, an area under the ROC curve of 0.77–0.78, and point totals that range between 0 and 12 [5]. For example, a 52 year-old non-smoking woman with small waist circumference would receive a score of 0 (indicating very low risk), while a 65 year old man with a large waist circumference, a brother with CRC, and a 30 pack-year history of cigarette smoking would receive a score of 11 (indicating high risk). While the risk index has potential to benefit both patients and providers, it is aimed primarily at providers, to help facilitate discussions about which test is most appropriate given patient risk for advanced neoplasia.

Current screening guidelines vary from country to country and within the U.S., among the different guideline organizations. In Europe, most countries use FIT as the primary screening test, reserving colonoscopy for persons with occult blood in the stool and for those at high-risk due to family history. The risk prediction tool would have less utility in these countries. However, some European countries (e.g., Poland and Germany) screen primarily with colonoscopy, as is done in the U.S. The risk prediction tool may have greater utility in these countries, as it would identify a sizeable subgroup with low risk for advanced neoplasia for which screening with FIT would be most appropriate. In the U.S., any of several tests (including FIT, high-sensitivity guaiac-based fecal blood tests, the multi-target stool DNA test, computed tomographic colonography, sigmoidoscopy, and colonoscopy) is recommended by the U.S. Preventive Services Task Force with no preference for any single test [4]. This lack of preference arises from absence of direct comparative evidence between or among the several tests [23], and because of the need to consider the tradeoffs among the tests for any individual person. Providers in this study acknowledged the potential of the risk prediction tool to help initiate the discussion about CRC screening so that the tradeoffs among tests could be considered within the context of patient risk for advanced neoplasia.

The preference-sensitive nature of CRC screening methods makes discussions about CRC highly appropriate for shared decision-making [24]. Shared decision-making allows providers and patients to share information, express preferences, discuss different options, and ultimately agree on a plan [25, 26]. Shared decision-making is foundational to patient-centered care [26, 27] and has been associated with positive outcomes, including better engagement in care and higher treatment adherence [28, 29]. Thus, engaging patients in shared decision-making about CRC screening has the potential to increase uptake by fostering patient engagement and adherence to a screening plan. Not surprisingly, several guidelines support shared decision making for cancer screening [1, 2, 30].

The importance of shared decision-making can be seen in Helsingen and colleagues’ recommendations for screening. These authors suggest that screening with FIT, sigmoidoscopy or colonoscopy should occur for those whose 15-year risk for CRC is 3% or greater [30], illustrating the importance of using risk to determine whom (and perhaps how) to screen. The risk prediction tool used in this study provides the current risk of advanced neoplasia as an alternative risk for providers and patients to consider: for very low- and low-risk patients, a FIT-based screening strategy would seem most appropriate, while colonoscopy would seem most appropriate for high-risk persons. Those at intermediate-risk could look to other factors (cost, burden, potential for benefit, preference for a longer versus shorter interval for re-testing) to help decide which test is best for them. The need to weigh all of these factors, especially for those at intermediate risk, presents an important opportunity for patients and their providers to engage in shared decision-making.

Since the risk prediction tool was designed primarily to help providers frame a discussion about CRC screening with their patients, we were not surprised to find that patient understanding required a detailed explanation from the interviewer, which may not be feasible in the clinical setting. A slender body of literature on this topic indicates that patients often have difficulty understanding risk [31]. Weinstein and colleagues studied patients who used a computer program that provided personalized information about 20-year risk of developing colorectal cancer [31]. Patients overestimated both absolute and relative risks, and nearly half did not accept personalized feedback as correct [31]. However, risk prediction linked to a specific behavioral prescription (e.g., colonoscopy or FIT) has been identified as more effective for presenting risk scores to patients [32].

Our results suggest that the tool is acceptable to patients and PCPs, with both groups supporting its potential to improve screening uptake. For use in daily practice, PCPs preferred the tool to be part of the EMR or in hard copy with patient-specific data and risk estimate included. Either format could or could not include a suggested screening test (e.g., stool blood testing for low-risk, colonoscopy for high-risk). Such information may be required to optimize the tool’s format/presentation and to align it with providers’ preferences. These findings will be incorporated into a clinical trial that will test whether the risk prediction tool improves uptake, test choice or both.

This study has limitations. First, interviews were conducted in two hospital systems with unique populations: a safety net hospital and a VA medical center, both within an academic environment. The results would not necessarily apply to other settings. Second, while the PCPs’ interviews were rich and detailed, patient interviews did not yield the same level of detail, limiting conclusions that can be drawn from the patients’ perspectives. Third, social desirability bias may have led some patients to overestimate understanding of the tool. Finally, participants’ responses (especially PCPs) could have been influenced by perceptions that the tool was developed by members of the study team, despite interviews being conducted by members unassociated with tool development. However, it is unlikely that these possible perceptions had a marked influence on the data, since PCPs identified negative perceptions of the tool. Use of reflexivity and negative case analysis during the data analysis process also helped to mitigate this potential limitation.

## Conclusions

With limitations in mind, this study suggests that using a risk prediction tool to help personalize CRC screening may be acceptable and appealing to both patients and PCPs and thus has potential for successful implementation. This is important because up to 35% of eligible patients are not current with CRC screening, despite its ability to reduce both incidence and mortality from CRC. Next steps include optimizing the tool’s presentation, incorporating feedback from the current study (e.g., helping PCPs to understand the data the tool is based on), integrating the tool into clinical workflow, and testing its ability to improve the uptake of CRC screening in clinical practice. If uptake is improved through greater use of non-invasive testing, it will be important to assess adherence over time for repeat non-invasive testing, and to determine whether the tool improves screening efficiency by targeting high-risk persons for colonoscopy.

## Supplementary information

**Additional file 1.** Qualitative Interview Guide.

## Data Availability

The datasets generated and/or analyzed during the current study are not publicly available as individual privacy could be compromised due to the nature of qualitative data.

## Abbreviations

CRC: Colorectal cancer
FIT: Fecal immunochemical test
PCP: Primary care providers
