# Supplementary material for: A risk prediction tool for colorectal cancer screening: a qualitative study of patient and provider facilitators and barriers
Source: BMC Fam Pract. 2020 Feb 26;21:43. doi: 10.1186/s12875-020-01113-0 (PMC7045431; doi:10.1186/s12875-020-01113-0)
Supplement: Supplementary file 1 — Additional file 1. Qualitative Interview Guide. [file 12875_2020_1113_MOESM1_ESM.zip › Patient Interview GuideR2.docx]

**Draft Patient Interview Guide**

Intro:

- Introduce and thank; tell how long (no more than 30 minutes)
- Tell subject what you’re going to tell them
  - Some questions about colon cancer testing and how you and your doctor / provider communicate
  - Introduce a new tool to estimate a person’s chances of having either colon cancer or precancerous polyps in the colon
  - Get reaction to and ask questions about the tool.

**Domain: History**

- Have you ever been tested for colon cancer?
  - If yes: when? Why did you get tested? (If they say because their doc recommended, ask: Were there any other reasons?)
    - What kind of test was it (e.g., colonoscopy, stool test, etc.)?
  - If no: Why not? (Probes: time, not wanting to go through a colonoscopy)
    - What would make you get tested? (probes: close friend or relative with CRC, fear, “because I’m supposed to”)

**Domain: Physician-Patient Communication**

- Have you and your doctor talked about having a test for colon cancer? (If this is already known from above questions, and they have discussed, ask: How have you and your doctor talked about having a test for colon cancer?)
  - If yes: How did that conversation go? (Probes: Has he/she brought it up recently? If so, what did you say?)
  - Did your doctor talk with you about the different ways you can get tested for colon cancer? What did he/she say? What was your reaction?
- (Ask if not covered above) If your doctor recommended a colonoscopy, what would you do? Explain.
- (Ask if not covered above) If your doctor recommended a test checking for blood in your stool, instead of a colonoscopy, would you be OK with that? (Explain).

**Domain: Knowledge/Barriers**

- Do you plan to have a (or another) colon cancer test any time soon?
  - If no, what is the biggest reason why you haven’t been tested? What would change your mind?
  - If yes, when? Is it scheduled?
- What do you know about how colon cancer screening is done?
  - (If they only talk about colonscopy): Did you know there’s at least one other test available? (RA: explain that it’s a test for blood in the stool. It is recommended every year, which is different from colonoscopy, which is every 10 years. Both tests are about equal for detecting cancer.)
  - Did you know this before? Does this information change how you think about getting tested for colon cancer? Why (not)?

**Domain: Risk Tool**

- Intro: There is a new aide available to help estimate your risk of having colon cancer or precancerous polyps (most of the risk is for the polyps). For patients at very low or low risk of colon cancer or precancerous polyps, a test that is simpler than colonoscopy could be recommended. For patients at high risk, colonoscopy would be recommended.
- TAKE PATIENT THROUGH THE SLIDES
- How easy is it to understand this tool?
  - (Consider “quizzing” the patient with a question or two.)
  - If not easy: what could make it easier to understand?
  - What could be clearer?
- How would you feel if your doctor used this tool to help find the best colon cancer test for you?
  - If you were “low risk” and your doctor recommended a stool test every year instead of colonoscopy, would that be okay? (Probes: Would you be comfortable with a stool test? Would you be relieved that you wouldn’t need a colonoscopy?)
- Is there anything else about this tool or about colon cancer testing that you’d like to share before we end the interview?

**End of Interview**
