# Supplementary material for: A risk prediction tool for colorectal cancer screening: a qualitative study of patient and provider facilitators and barriers
Source: BMC Fam Pract. 2020 Feb 26;21:43. doi: 10.1186/s12875-020-01113-0 (PMC7045431; doi:10.1186/s12875-020-01113-0)
Supplement: Supplementary file 1 — Additional file 1. Qualitative Interview Guide. [file 12875_2020_1113_MOESM1_ESM.zip › Provider Interview GuideR2.docx]

**PCP Interview Guide**

**Domain: Decision Making/Pt-provider Communication**

- How do you bring up the topic of CRC screening with your patients? What do you typically tell them?
  - What options if any do you present for screening? Why do you present different options (or why not)? Does this vary based on the patient? If so, how?
  - If a patient refuses colonoscopy, do you offer other screening tests? (if no, why not) (If yes) How do patients usually respond when you offer alternative tests? (Probe for specifics if necessary)
  - Do you discuss risks of screening? (If no, why not?) What do you say about risks?
- When you choose a screening test for your patients, do you tend to recommend the same test for everyone?
  - Do you take a patient’s individual risk into consideration? How likely are you to ask the patient for his/her preference and use this to inform your recommendations?
    - How is the final decision made (i.e., do you involve the patient in the decision making?)?
- When patients choose not to be screened for CRC what are their reasons? How do you address these?

**Domain: Use of the Risk Model**

- (Assuming an average-risk patient with no specific screening preferences) What do you think about this risk index?
- If this tool were integrated into the Electronic Health Record, how likely would you be to use it to discuss CRC screening with your patients?
  - (if likely) What about the tool appeals to you?
  - (if not likely) What makes you unlikely to use this tool? (would need to probe into responses)
- What do you think about having a nurse or medical assistant input the data for patients and provide you with the results?
- If this tool were developed into a software app, would you use it? If no, why not?
- Which of the two modalities – a software app or integration into the electronic medical record - has greater appeal to you? (If EHR): How would you like to see this tool integrated into the EHR?
- Do you think a tool like this would increase uptake of CRC screening by patients who are not up to date with their CRC screening? Why or why not?
- Could using a tool like this help you in your practice? If so, how? (probes: aids clinical judgment, provides understandable risk estimate, saves time) If NOT, why not?
- What are the disadvantages of a tool like this? (Ask the following probes after the interviewee’s open response: Are you concerned about: missing a cancer, getting sued, inadequate validation of the tool, not trusting the model, the tool taking away your clinical judgment, too time-consuming to use)
